# Quantum-inspired cascaded discrete-time quantum walks with induced chaotic dynamics and cryptographic applications

**DOI:** 10.1038/s41598-020-58636-w

**Published:** 2020-02-06

**Authors:** Ahmed A. Abd El-Latif, Bassem Abd-El-Atty, Mohamed Amin, Abdullah M. Iliyasu

**Affiliations:** 1 Mathematics and Computer Science Department, Faculty of Science, Menoufia University, Shebin El-Koom, 32511 Egypt; 2 Department of Cybersecurity, College of Computer Science and Engineering, University of Jeddah, Jeddah, 23890 Saudi Arabia; 3 School of Computer Science and Technology, Harbin Institute of Technology, Harbin, 150080 China; 4 Electrical Engineering Department, College of Engineering, Prince Sattam Bin Abdulaziz University, Al-Kharj, 11942 Saudi Arabia; 5 School of Computing, Tokyo Institute of Technology, Yokohama, 226-8502 Japan; 6 School of Computer Science and Technology, Changchun University of Science and Technology, Changchun, 130022 China

**Keywords:** Computer science, Information technology

## Abstract

Designing efficient and secure cryptosystems has been a preoccupation for many scientists and engineers for a long time wherein they use chaotic systems to design new cryptosystems. While one dimensional (1-D) chaotic maps possess powerful properties compared to higher dimension ones, they are vulnerable to various attacks due to their small key space, chaotic discontinuous ranges, and degradation in chaotic dynamical behaviours. Moreover, when simulated on a computer, every such chaotic system produces a periodic cycle. Meanwhile, quantum random walks exhibit the potential for deployment in efficient cryptosystem design, which makes it an excellent solution for this problem. In this context, we present a new method for constructing substitution boxes (S-boxes) based on cascaded quantum-inspired quantum walks and chaos inducement. The performance of the proposed S-box scheme is investigated via established S-box evaluation criterion and outcomes suggest that the constructed S-box has significant qualities for viable applications information security. Further, we present an efficient scheme for pseudo-random numbers generation (PRNG) whose sustainability over long periods remedies the periodicity problem associated with traditional cryptographic applications. Furthermore, by combining the two mechanisms, an atypical image encryption scheme is introduced. Simulation results and analysis validate that the proposed image encryption algorithm will offer gains in many cryptographic applications.

## Introduction

Chaotic systems have attracted a great deal of attention across different scientific and engineering disciplines, especially in designing new cryptosystems and cryptanalysis. A chaotic system is an evolution map of a deterministic dynamical system that reconstructs the state of a system *S*_0_ to a new state *S*_1_ depending on the initial state of *S*_0_, a control parameter *C*, and time *T*^1^. Chaotic maps exhibit the desired properties of ergodicity, unpredictability, and sensitivity to their control parameter(s) and initial value(s) that satisfy the requirements for cryptosystem confusion-diffusion properties^2–4^. In fact, an inappropriate initial control parameter of a chaotic system can lead to non-chaotic behaviours, which implies the reduction in nonlinearity levels as well as circumvention of insecurity pitfalls^5,6^.

Currently, chaotic dynamical systems play a vital role in designing modern cryptographic applications, such as constructing S-boxes, generating pseudo-random numbers, designing image encryption algorithms and so on^7–16^, which are based on the unproven assumptions pertaining to computational complexity and that their constructions are based on mathematical models. However, with the development of quantum technologies, some of these traditional security mechanisms, and cryptographic applications may be effortlessly violated and abused^17–19^.

Among the computational models developed in quantum computation, quantum walks (QWs), which is a universal model of quantum computation that has been traditionally employed to develop modern quantum algorithms^20,21^. While physical quantum computing hardware are as yet unavailable, quantum inspired frameworks provide platforms for simulating pseudo-quantum algorithms, which, within the limits of bounds imposed by the capability of digital computers, can to execute some of the quantum mechanical properties ascribed for the potency of quantum computation^22–24^. Moreover, based on the rationale that computation of the position probability distribution of a quantum walker requires computation of probabilities of frequencies (i.e. the number of detections at a given graph vertex divided by the total number of detections). This requires sufficient number of repetitions of the experiment in order to retrieve the probability distribution. Among others, this has motivated the use of quantum-inspired discrete-time quantum walks have been presented as viable resources useful in designing chaotic system for image encryption algorithms^25–29^. This procedure allows us to consider a quantum-inspired discrete quantum walk *Q* as a nonlinear mapping $Q:H\mapsto P$ where *H* is a Hilbert space in which the walker exists and *P* is a set of probability distributions. At this juncture, we note that our notion of a quantum-inspired approach implies use of probability distribution of a quantum walk obtained from numerical simulations using digital resources.

The nonlinear behaviour of quantum-inspired walks described above together with the deterministic nature of state growth via unitary operators as well as the high sensitivity of quantum walks to initial conditions support the treatment of quantum-inspired discrete quantum walks as discrete-time and discrete-value chaotic systems^25,26,30^.

Inspired by the excellent dynamical properties of quantum walks, the limitations of traditional cryptosystems can be ameliorated via design and construction state-of-the-art techniques for effective information security applications. In addition to other benefits, a main contribution of this study is to explore the integration of quantum-inspired of quantum walks into traditional cryptographic applications. Hence, we present a bi-level cascaded quantum walks protocol as a quantum-inspired random number generator with chaos inducement. The performance of the proposed S-box scheme is investigated using established criterions, results of which suggest that the constructed S-box is viable for multifaceted applications in information security. Similarly, the analyses of the proposed PRNG suggest its efficiency in generating sequences that remedy the periodicity problem associated with traditional cryptographic applications. Finally, we deploy the dual cascade quantum walks and chaos systems for applications in image encryption. Throughout, simulation-based validation is used to assess the performance of the proposed scheme. Outcomes from our applications for S-boxes construction, pseudo-random number generation, and image encryption validate the choice of cascaded quantum walks and chaos inducement for various cryptographic applications. At this point we clarify that this study is focused on exploiting properties of quantum walks for use in a quantum-inspired setting for potential applications in traditional cryptography. Hence, the quantum mechanical implementation of quantum walks is deemed outside the purview of this present work. Nevertheless, we enrich our bibliography by including interesting studies on such implementation^19,20,31–40^ from where interested readers can obtain further details.

## Results

### S-box construction

Designing powerful S-boxes is an important critereon for realisation of secure cryptosystems and it is a major component of nonlinear transformations, which are the fulcrum of confusion and diffusion analysis for assessing well-designed ciphers^41^. Therefore, designing S-boxes based on secure mechanisms plays an important part in modern cryptographic tasks^42,43^. Consequently, it is widely investigated. For example, in a recent effort EL-Latif *el al*.^30^ explored construction of secure S-boxes based on one-dimensional two-walker QWs on a circle. Inspired by the potency of quantum technologies, in this section, we propose a mechanism to augment some shortcomings of standard S-box construction and integrate our upgraded design into a cascaded QW and chaos inducement system for designing efficient cryptographs.

The following steps outline the construction of an M-length S-box.

**Step 1:** Choose initial seed for *x*_0_ and a value for the control parameter *λ*, to iterate the logistic-sine map over *N* times needed to generate sequence $\{{X}_{i}\}$.

**Step 2:** Choose initial conditions and key parameters ($v,t,{\alpha }_{1},{\alpha }_{2},{\beta }_{1},{\beta }_{2}$) for running QWs on a circle with *v* vertices to produce a probability matrix ${P}_{v\times v}$, where *v* is odd number, ${\alpha }_{1},{\alpha }_{2},{\beta }_{1},{\beta }_{2}\in [0,\pi ]$ and t is the number of steps for running QWs. Hence, the coin operator $\hat{C}$ constructed by the key parameters ${\beta }_{1}$ and ${\beta }_{2}$, while the initial states of the two walkers are ${H}_{C1}=\,\cos \,{\alpha }_{1}|0\rangle +\,\sin \,{\alpha }_{1}|1\rangle$ and ${H}_{C2}=\,\cos \,{\alpha }_{2}|0\rangle +\,\sin \,{\alpha }_{2}|1\rangle$, respectively.

**Step 3:** Resize *P* to *QW*_*N*_, where *N* is the number of iterated chaos map. Here, we recall that mathematically no error arises from scaling a matirix with fixed dimentions several times. Targeting such a property, in this step, we make use of the bicubic interpolation resizing^44^, which has zero error during the scalling process etc. This attribute allows it to accommodate prolonged iterations in the chaos map generation.

**Step 4:** Convert the sequences $\{{X}_{i}\}$, and $\{Q{W}_{i}\}$ into integer values via Eqs. (1) and (2).

1 $$S{X}_{i}=|fix({X}_{i})\times {10}^{8}|\,{\rm{mod}}\,M$$

2 $$SQ{W}_{i}=|fix(Q{W}_{i})\times {10}^{12}|\,{\rm{mod}}\,M$$

**Step 5:** Perform the bitwise XOR operation on the sequences $\{S{X}_{i}\}$, and $\{SQ{W}_{i}\}$ to produce the sequence $\{{S}_{i}\}$ with range from 0 to *M*-*1*.

**Step 6:** Collate the first *M* dissimilar elements from the sequence $\{{S}_{i}\}$ to construct the desired S-box.

The performance of the S-box construction technique is investigated using a workstation equipped with Intel® core™ i5-2450M CPU 2.5 GHz and 6 GB RAM with a preinstalled MATLAB software. The initial values for running QWs are set as $v=17$, $t=57$, ${\alpha }_{1}=0$, ${\alpha }_{2}=\pi /2$, ${\beta }_{1}=\pi /6$, ${\beta }_{2}=\pi /6$, while initial values used to iterate the logistic-sine map are set as ${L}_{0}=0.4$, $\lambda =3.82$.

The constructed 16 × 16 S-box costructed based on the aforesaid initial conditions and control parameters is presented in Table 1, while Table 2 provides comparison of the performance of the constructed S-box alongside those some published schemes alongside the proposed one in terms of standard parameters of strict avalanche (SAC), nonlinearity, bit independence (BIC), as well as differential (DP) and linear (LP) approximation probabilities.

**Table 1:** 16 × 16 S-box constructed via proposed scheme.

205	51	93	103	62	198	199	224	149	114	75	48	132	102	142	125
204	173	253	23	180	65	245	50	208	118	117	121	156	38	152	138
193	128	243	127	105	96	4	154	76	251	196	169	95	120	190	98
211	179	175	188	81	219	41	84	218	195	200	153	248	209	36	207
30	157	183	67	143	194	135	133	64	236	3	33	254	86	49	79
227	240	249	104	163	250	115	78	74	68	178	17	162	159	12	139
18	11	164	191	61	235	87	181	222	113	108	226	106	221	37	241
29	177	174	2	6	202	99	92	184	158	172	171	0	242	215	28
40	5	189	214	206	24	165	110	26	155	246	14	111	230	237	52
69	182	59	122	197	231	116	234	56	35	167	13	101	126	27	210
42	119	91	60	147	216	166	89	203	112	53	55	71	124	39	130
85	31	72	19	45	185	168	150	186	90	22	212	1	15	107	141
140	144	77	151	131	232	238	247	136	217	233	58	21	145	88	225
129	228	201	146	255	46	32	7	44	82	70	20	97	43	83	134
187	10	239	34	47	137	109	229	252	213	161	94	123	170	160	63
80	220	57	148	9	16	54	25	100	244	73	66	8	176	192	223

**Table 2:** Evaluation of the performance of proposed S-box construction alongside other methods.

S-box scheme	BIC-NL	Nonlinearity	BIC-SAC	SAC	LP	DP
Proposed	103.93	106	0.5023	0.4958	0.1250	0.0313
EL-Latif *et al*.^30^	103.70	106.25	0.5065	0.5037	0.1016	0.0391
Belazi *et al*. S-box^61^	103.78	105.50	0.4970	0.5000	0.1250	0.0468
Khan *et al*.^62^	103.07	103.25	0.4864	0.5151	0.1563	0.17187
Wang *et al*. S-box^63^	103.36	104.87	0.5017	0.4918	0.1328	0.0391
Tang *et al*. *et al*.^64^	103.00	105.00	0.5044	0.4971	0.1328	0.0391
Özkaynak *et al*.^65^	103.14	104.62	0.4942	0.4982	0.1406	0.0391
Belazi *et al*.^66^	103.80	105.25	0.4996	0.4956	0.1562	0.0391
Hussain *et al*.^67^	104.29	103.25	0.5021	0.5056	0.1289	0.04609

### PRNG generator

Pseudo-random number generation (PRNG) plays a fundamental role in creating powerful cryptographic schemes and, as such, they attract a great deal of attention from many cryptographers and engineers. The key feature of PRNG is to provide long streams of numbers embedded with randomness features. PRNG has a vital impact on the robustness of cryptographic tasks and in mitigating attempts to violate, tamper with, or regenerate the secret information being protected. The common approach employed in designing PRNG generators is based on using chaos maps, which is a simple (in terms of definition), yet disorienting approach intended to circumvent infractions to sensitive information^9,11^. Previous efforts, such as^45^, profit from the utility of quantum walks to overcome established limitations of traditional chaos maps. Furthermore, Yang *et al*.^45^ proposed a novel PRNG mechanism based on quantum walks.

**Figure 1:**
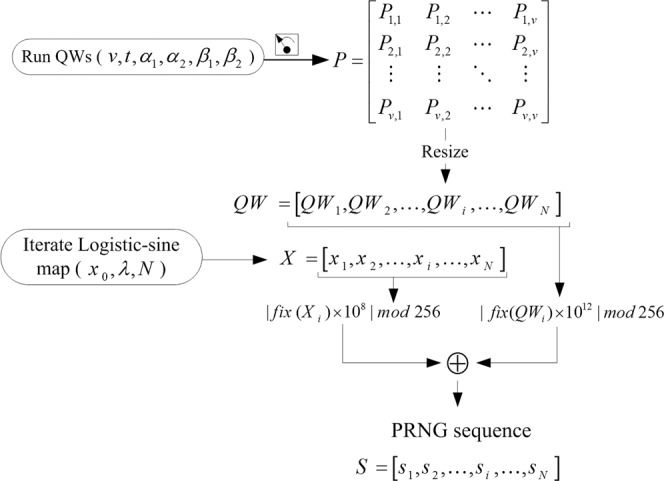
Outline of the proposed PRNG sequence generation mechanism.

Motivated by the effort in^45^, in this section, we discuss our proposed mechanism for PRNG sequence generation whose outline is presented in Fig. 1 and execution is accomplished via the five steps enumerated in the sequel.

**Step 1:** Select initial seed for ${x}_{0}$ and a value for the control parameter $\lambda$, to iterate the logistic-sine map over *N* times needed to generate sequence $\{{X}_{i}\}$.

**Step 2:** Select initial conditions and key parameters ($v,t,{\alpha }_{1},{\alpha }_{2},{\beta }_{1},{\beta }_{2}$) for running QWs on a circle with *v* vertices to produce a probability matrix ${P}_{v\times v}$, where *v* is odd number, ${\alpha }_{1},{\alpha }_{2},{\beta }_{1},{\beta }_{2}\in [0,\pi ]$ and t is the number of steps for running QWs. Hence, the coin operator $\hat{C}$ constructed by the key parameters ${\beta }_{1}$ and ${\beta }_{2}$, while the initial states of the two walkers are ${H}_{C1}=\,\cos \,{\alpha }_{1}|0\rangle +\,\sin \,{\alpha }_{1}|1\rangle$ and ${H}_{C2}=\,\cos \,{\alpha }_{2}|0\rangle +\,\sin \,{\alpha }_{2}|1\rangle$, respectively.

**Step 3:** Resize $P$ to $Q{W}_{N}$, where *N* is the number of iterations for the chaos map as well as the length of desired PRNG sequence.

**Step 4:** Convert the sequences $\{{X}_{i}\}$, and $\{Q{W}_{i}\}$ into integer values as follows:

$$S{X}_{i}=|fix({X}_{i})\times {10}^{8}|\,{\rm{mod}}\,256$$

$$SQ{W}_{i}=|fix(Q{W}_{i})\times {10}^{12}|\,{\rm{mod}}\,256$$

**Step 5:** Perform bitwise XOR operation on the sequences $\{S{X}_{i}\}$, and $\{SQ{W}_{i}\}$ to generate a PRNG sequence, *S* of length *N*.

To investigate the randomness property of the generated PRNG sequence S, we applied NIST SP 800-22 specified tests. These tests comprise of fifteen (15) assessments that are performed on a generated sequence of 10^6^ bits length. We used the same initial values and control parameters for constructing S-box to generate the PRNG sequence whose results are presented in Table 3. As seen therefrom, the sequence generated via the proposed mechanism excelled in all tests carried out; thus, confirming its utility across various cryptographic applications.

**Table 3:** Results for NIST SP 800-22 tests.

Test-Name	P-Value	Result
Overlapping templates	0.215108	Passed
No overlapping templates	0.079004	Passed
DFT	0.304052	Passed
Frequency	0.291883	Passed
Block-frequency	0.693686	Passed
Universal	0.612656	Passed
Rank	0.058737	Passed
Long runs of ones	0.137157	Passed
Runs	0.384907	Passed
Serial 1	0.914512	Passed
Serial 2	0.971079	Passed
Random excursions variant x = 1	0.506620	Passed
Random excursions x = 1	0.125622	Passed
Linear complexity	0.107102	Passed
Cumulative sums (reverse)	0.065686	Passed
Cumulative sums (forward)	0.520534	Passed
Approximate entropy	0.012095	Passed

### Application of proposed cascade protocol in image encryption

The intuition to utilise chaos systems in image encryption is not new, including many employing one-dimensional or higher dimension chaotic systems to generate a sequence of random numbers for construction of a cipher image that have been broached in^12–16^. However, most of these approaches produce images that are vulnerable to various attacks due to their narrow key-space allowance and imprecise mathematical construction. Consequently, to ameliorate this, some interesting image encryption algorithms based on the dynamical properties of QWs were proposed in^25,26^ and^27^.

In this section, we exploit the potency of quantum computing technologies to ameliorate some established shortcomings inherent to existing chaos systems. Our proposed image encryption technique utilises the S-box construction and PRNG sequence generation methods presented in earlier sections of this study to substitute and permutate each pixel of a plain image and construct its encrypted version. These procedures and their perfomance analysis are further elucidated in the remainder of this section.

**Figure 2:**
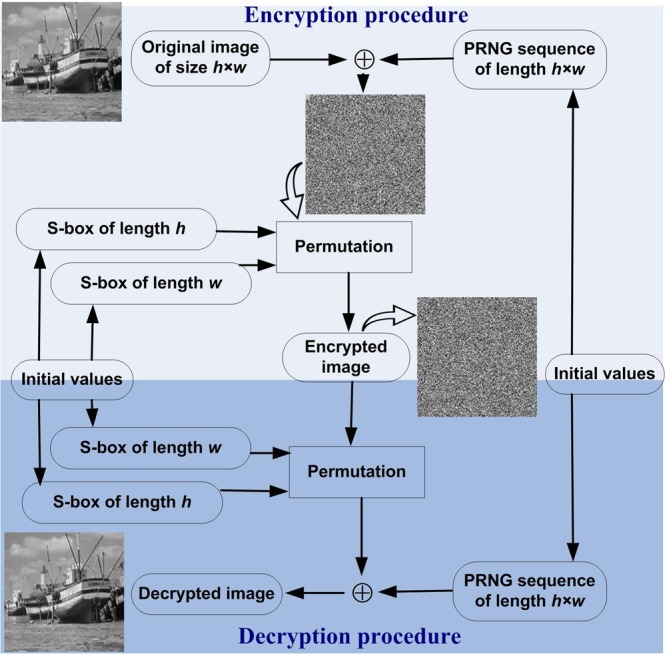
General framework for the proposed image encryption technique.

The general framework for the proposed image encryption technique is illustrated in Fig. 2, while the encryption procedures are outlined in the following steps.

1. Select initial values for generating two S-boxes *SH* and *SW* of lengths *h* and *w* respectively, where the size of the original image is *h* × *w*.
2. Select initial values for generating one PRNG sequence *K* of length *h* × *w* (or *h* × *w* × *3* for colour images) where the size of the original image is *h* × *w*.
3. Perform bitwise XOR operation on original image and matrix K to obtain an Xored image.
4. Permutate the Xored image using the constructed S-boxes as outlined in Algorithm 1.

**Algorithm 1:**
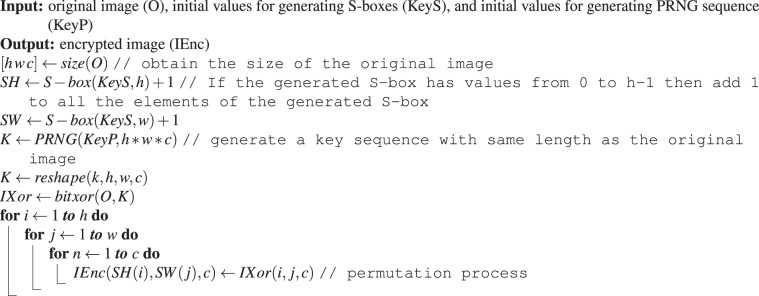
Image encryption algorithm.

### Performance analysis

**Figure 3:**
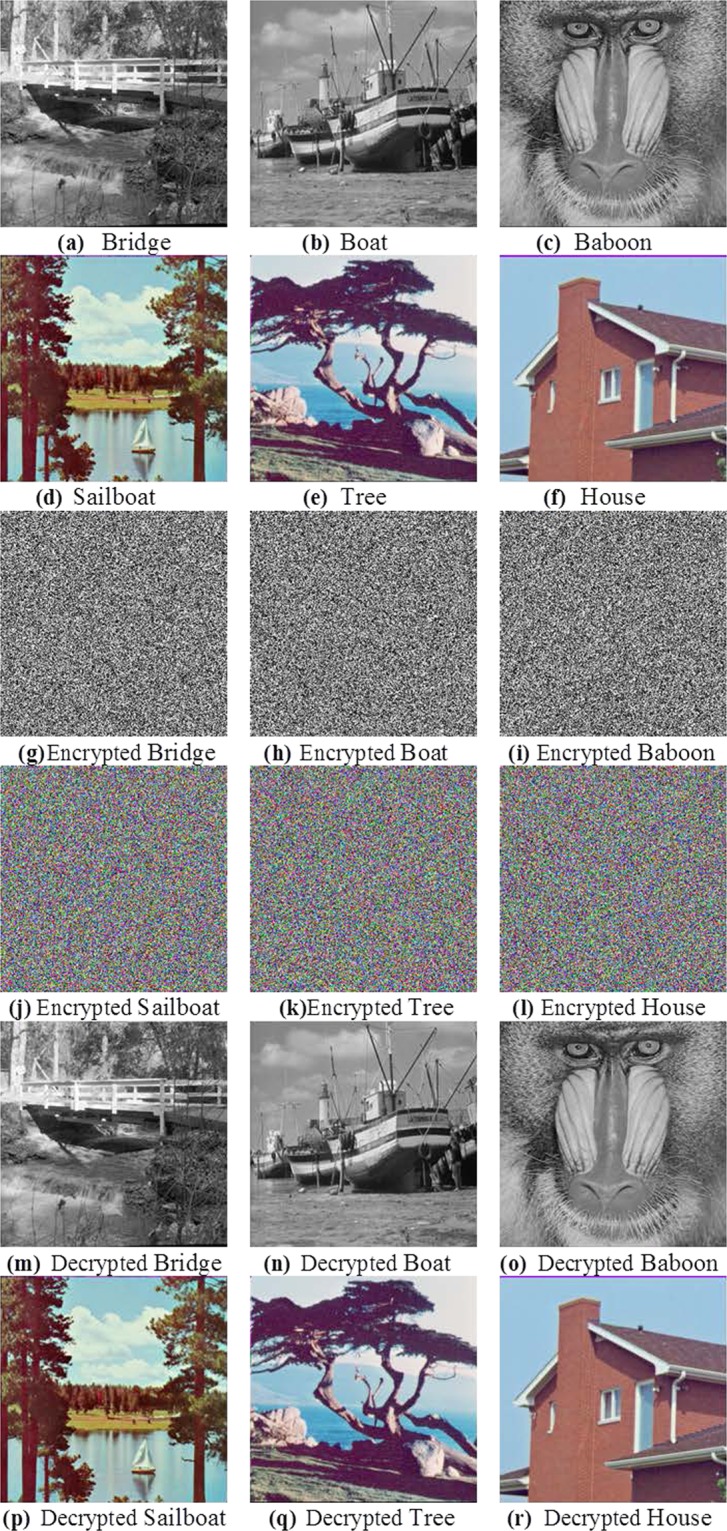
Test images (**a**–**f**), their encrypted (in (**g**–**l**)), and decrypted (in (**m**–**r**)) versions.

To validate the proposed strategy, we simulated implementation of the image encryption algorithm using a dataset comprising of three greyscale (Bridge, Boat and Baboon) and three colour images (Sailboat, Tree and House) sourced from the Signal and Image Processing Institute dataset^46^ and each of 256 × 256 dimensions. These test images are presented in Fig. 3(a–f). Initial values for running the QWs to construct S-boxes and generate PRNG sequences were set at $v=19$, $t=25$, ${\alpha }_{1}=0$, ${\alpha }_{2}=\pi /2$, ${\beta }_{1}=\pi /6$, ${\beta }_{2}=\pi /4$, while initial values used to iterate the logistic-sine map are set as ${L}_{0}=0.7524$, $\lambda =3.8245$.

The resulting encrypted versions of the test images are presented in Fig. 3(g–m) and based on the pairing of each original and encrypted image pair we undertook a retinue of statistical analysis whose results are presented and discussed in subsequent subsections.

#### Correlation of adjacent pixels

**Figure 4:**
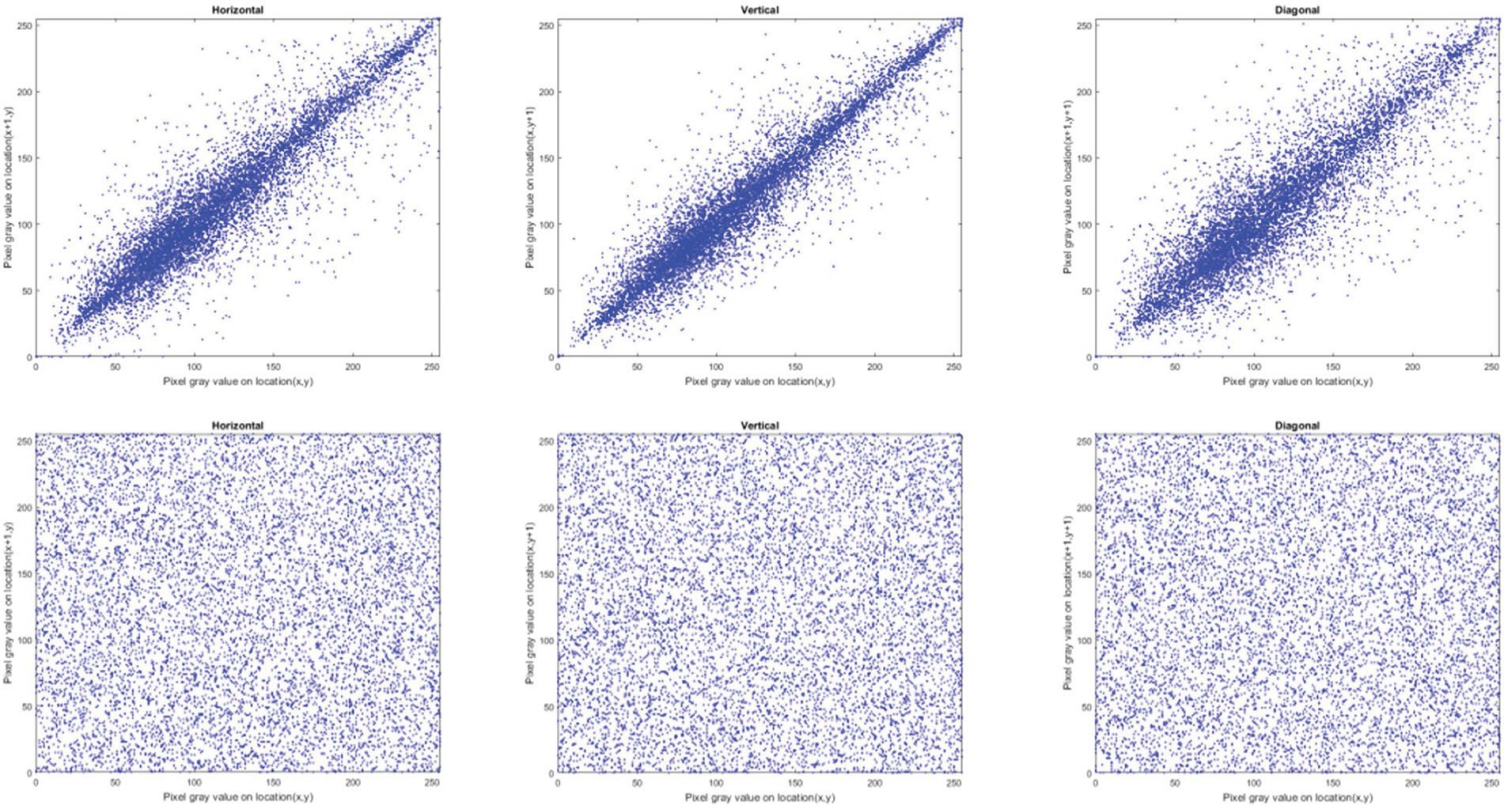
Correlation distribution for neighbouring pixel pairs along horizontal, vertical and diagonal directions for Bridge image in Fig. 3(a).

**Figure 5:**
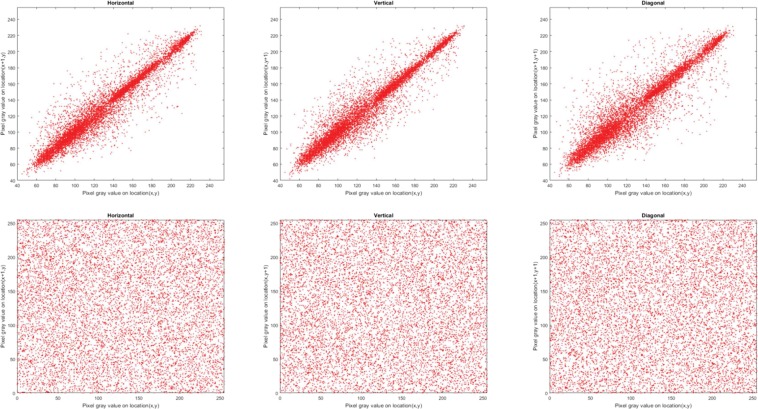
Correlation distribution for neighbouring pixel pairs along horizontal, vertical and diagonal directions for red channel of Sailboat image in Fig. 3(d).

**Figure 6:**
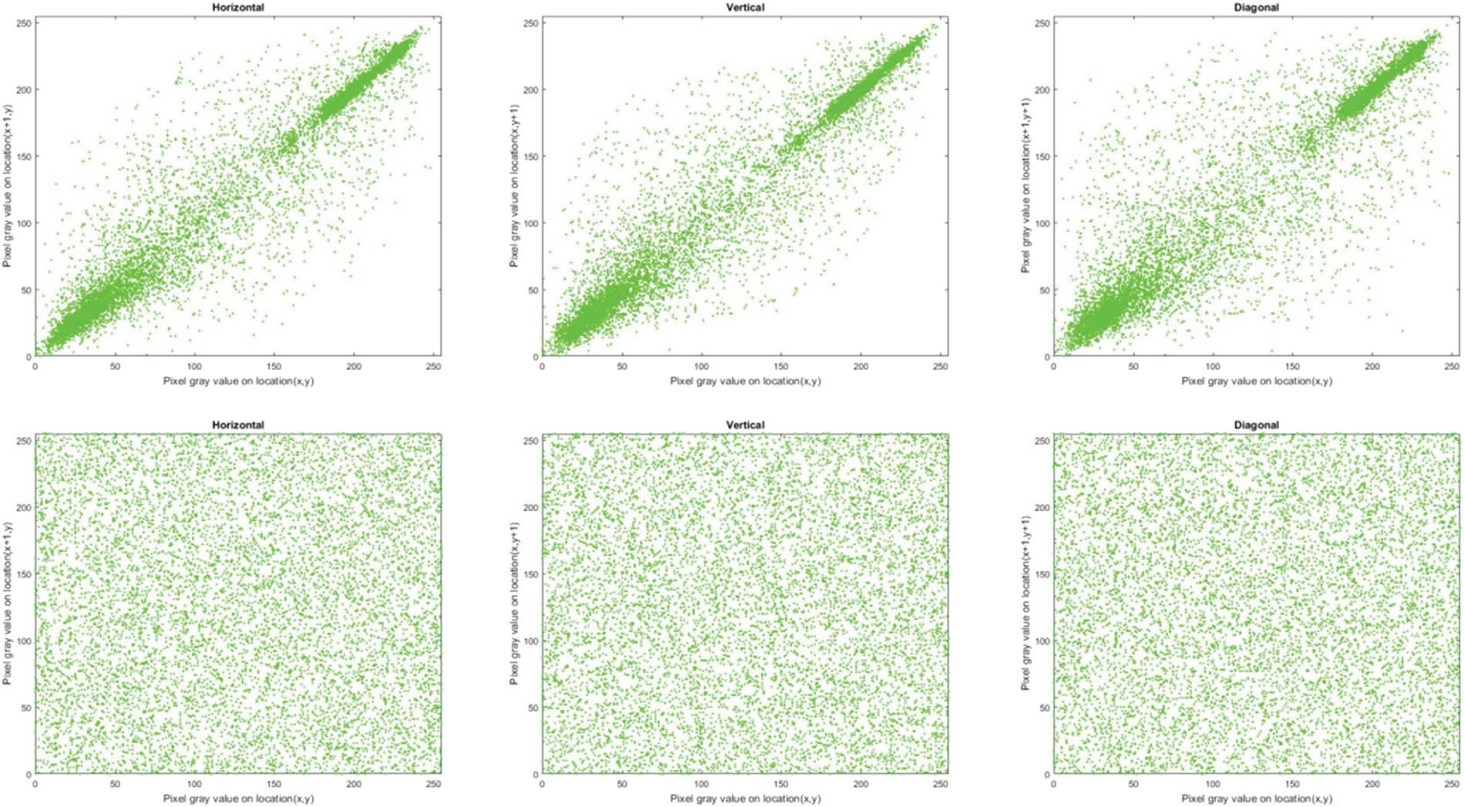
Correlation distribution for neighbouring pixel pairs along horizontal, vertical and diagonal directions for green channel of Sailboat image in Fig. 3(d).

**Figure 7:**
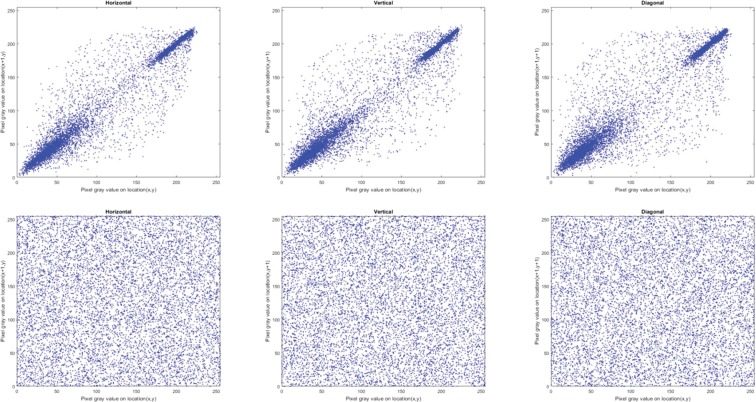
Correlation distribution for neighbouring pixel pairs along horizontal, vertical and diagonal directions for blue channel of Sailboat image in Fig. 3(d).

Correlation coefficient, *C*_*xy*_, is used to measure concordance between two adjacent pixels *X* and Y in an image. Theoretically, a pristine, i.e. unencrypted, image should have *C*_*xy*_ values close to 1 in each direction (horizontal, vertical and diagonal) whereas a well encrypted image should have values close to 0^47–49^. To compute *C*_*xy*_ for the encrypted and original images in each direction, we randomly selected 10,000 pairs of neighbouring pixels and used (3) to quantify their correlation.

3 $${C}_{xy}=\frac{{\sum }_{i=1}^{M}\,({x}_{i}-\bar{x})\,({y}_{i}-\bar{y})}{\sqrt{{\sum }_{i=1}^{M}\,{({x}_{i}-\bar{x})}^{2}\,{\sum }_{i=1}^{M}\,{({y}_{i}-\bar{y})}^{2}}}$$

where ${x}_{i}$ and ${y}_{i}$ are values of adjacent pixels and M is the total number of adjacent pixel pairs in each direction. Tables 4 and 5 present the values of *C*_*xy*_ for the encrypted and corresponding original images, where the encrypted images have *C*_*xy*_ values close to 0. The distribution of neighbouring pixel pairs in each direction of Bridge image are graphed in Fig. 4, while those for the R, G, and B channels of the Sailboat colour image are presented in Figs. 5, 6 and 7, respectively. The results in Tables 4 and 5 as well as those in Figs. 4, 5, 6 and 7 suggest that for the three pairs reported there is no relation between the encrypted images and their original versions.

**Table 4:** Correlation coefficients for adjacent pixel pairing for greyscale images (in Fig. 3(a–c)).

Image	Direction		
	Horizontal	Vertical	Diagonal
Original (Bridge)	0.9160	0.9416	0.8845
Encrypted (Bridge)	0.0002	0.0026	−0.0003
Original (Boat)	0.9436	0.9246	0.8811
Encrypted (Boat)	−0.0034	−0.0043	−0.0012
Original (Baboon)	0.8304	0.8776	0.7963
Encrypted (Baboon)	−0.0050	0.0001	0.0006

**Table 5:** Correlation coefficients for adjacent pixel pairing for colour images (in Fig. 3(d–f)).

Image	Direction								
	Horizontal			Vertical			Diagonal		
	R	G	B	R	G	B	R	G	B
Original (Sailboat)	0.9552	0.9555	0.9644	0.9582	0.9567	0.9606	0.9311	0.9249	0.9373
Encrypted (Sailboat)	−0.0003	−0.0082	−0.0003	−0.0022	−0.0020	0.0047	0.0013	0.0004	0.0010
Original (Tree)	0.9392	0.9485	0.9438	0.9584	0.9696	0.9615	0.9221	0.9339	0.9308
Encrypted (Tree)	−0.0029	−0.0048	−0.0023	−0.0013	−0.0012	−0.0050	−0.0007	0.0012	−0.0061
Original (House)	0.9357	0.9636	0.9764	0.9678	0.9812	0.9824	0.9107	0.9490	0.9641
Encrypted (House)	0.0027	−0.0081	−0.0009	−0.0023	0.0005	0.0030	−0.0040	0.0007	−0.0029

#### Pixel change rate

Another tool used to evaluate the effect of changing pixel values in an original image on its corresponding encrypted one is number of pixel change rate (NPCR), which is computed using (4).

4 $$NPCR=\frac{{\sum }_{i;j}\,D(i,j)}{M}\times 100 \% ,\,D(i,j)=\left\{\begin{array}{l}0\,if\,X(i,j)=Y(i,j)\\ 1\,if\,X(i,j)\ne Y(i,j)\end{array}\right.$$

where *M* denotes total number of pixels in the image. The fact that, as reported in Table 6, all the test images (in Fig. 3(a–f)) produced NPCR values of approximately 99.60% shows that the proposed encryption strategy is very sensitive to small changes in pixel values in the original image.

**Table 6:** NPCR test results.

Image	NPCR (%)
Bridge	99.63837
Boat	99.59717
Baboon	99.61395
Sailboat	99.61853
Tree	99.61294
House	99.60124

#### Histogram analysis

**Figure 8:**
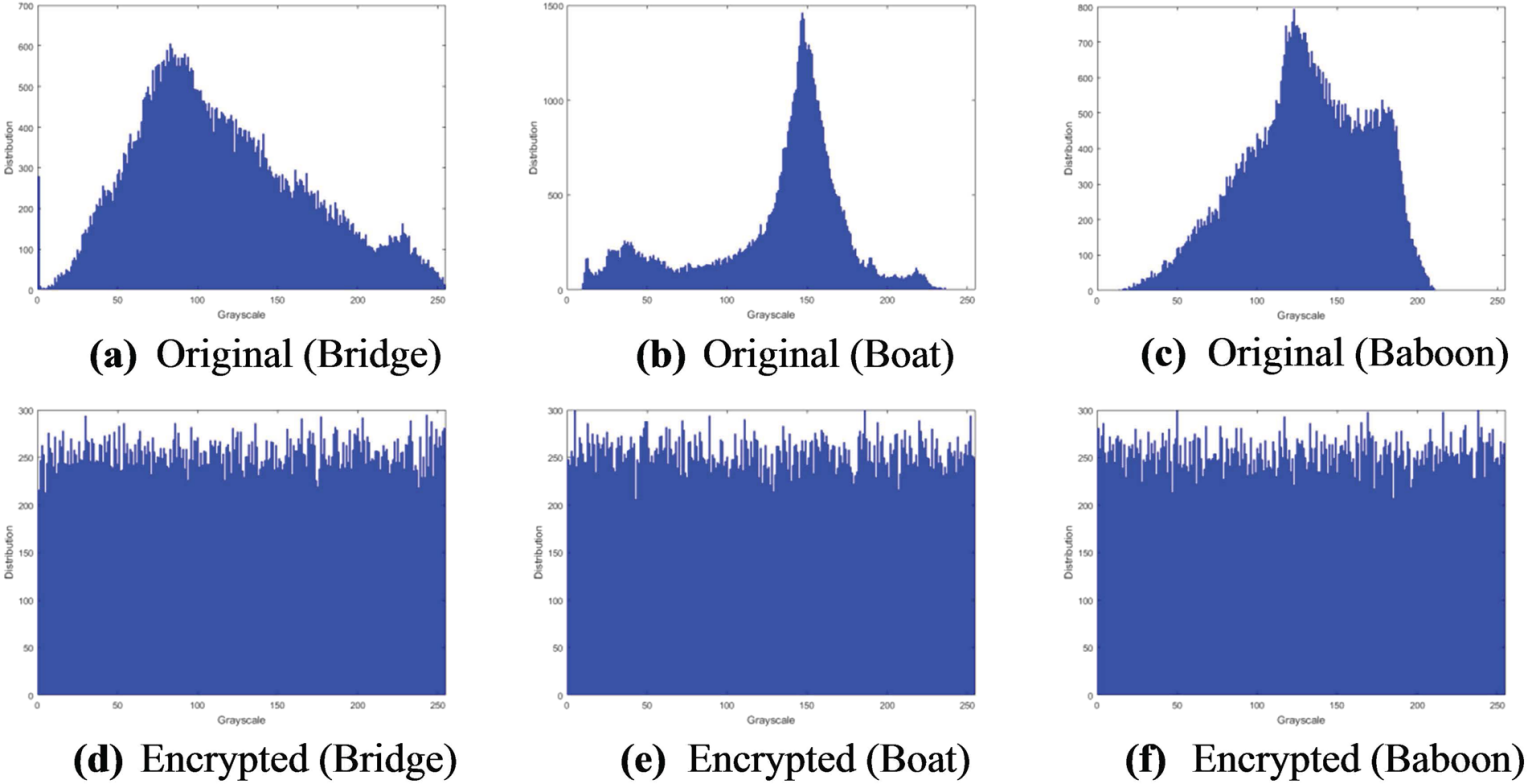
Histograms of original and encrypted greyscale images (in Fig. 3(a–c)).

**Figure 9:**
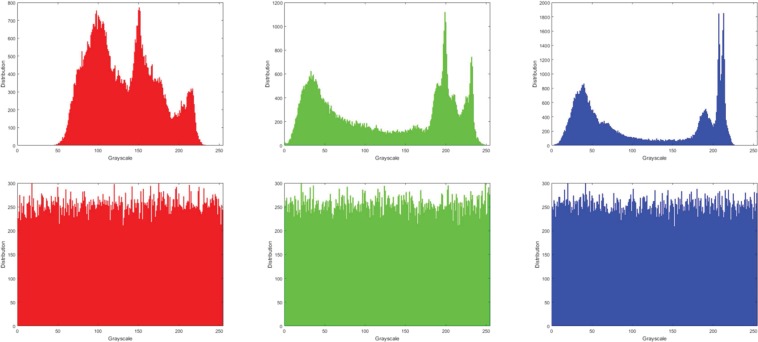
Histograms of original and encrypted R, G, and B channels of the Sailboat image (in Fig. 3(d)).

Histogram analysis is another widely used measure in image analysis that reflects the frequency distribution of pixel values in an image. A well-designed image encryption algorithm should have uniform histograms for different encrypted images, which is an indication of resistance against statistical attacks. Figures 8 and 9 present histograms for the original and encrypted versions of the greyscale images (in Fig. 3(a–c)) as well as the coloured colour Sailboat image in Fig. 3(d). Interpreting these plots, we deduce similarity in the distribution for the encrypted images. This is an affirmation that the encrypted images consist of flat-out noise. Meanwhile, the variability in the histograms of the original images indicate the presence of different levels of detail in those images. From the histogram analysis there is no relation between the encrypted image and its original one. Therefore, the proposed image encryption mechanism could resist histogram analysis attacks.

#### Information entropy

Information entropy, E(X), is an important tool to evaluate the efficiency of an image encryption algorithm. As expressed in (5), E(X) is a statistical measure of the distribution of pixel values for each level in an image.

5 $$E(X)=-\,\mathop{\sum }\limits_{i=1}^{{2}^{L}-1}\,p({x}_{i})\,{\log }_{2}\,(p({x}_{i}))$$

where $p({x}_{i})$ is the probability of obtaining ${x}_{i}$. Greyscale images have 2^8^ possible values based on which the ideal theoretical entropy value should be 8 bits^27^. Consequently, for an efficient encryption mechanism, the entropy value for the encrypted images should be close to 8. Table 7 presents the entropy values for the pristine and corresponding encrypted images used in our experiments (i.e. Fig. 3). As targeted, the information entropies for almost all the pairings is expected to be 8 bits (Table 7). This certifies the viability of the proposed algorithm to withstand entropy-based attacks.

**Table 7:** Information entropy of original and encrypted images.

Image	Original	Encrypted
Bridge	7.66847	7.99710
Boat	7.15866	7.99734
Baboon	7.22794	7.99729
Sailboat	7.35408	7.99727
Tree	7.18159	7.99700
House	6.40067	7.99704

#### Key space analysis

Theoretically, quantum-inspired quantum walks have an infinite key space^25,26,45^, but due to the finite precision of digital computers, the key space is limited. Therefore, the key space size is evaluated relative to the 10^−16^ precision of digital computers, which is acceptable for quantum insipired numerical simulation of quantum walks on digital computers^50,51^. However, it is highly unrealistic for actual physical implementation of a quantum walk, which would be the goal of future quantum technologies. Nevertheless, such simulation would suffice for classical-based quantum inspired simulation of our proposed random number generator.

A well-designed encryption algorithm should have adequate key space allowance to withstand brute-force and other attacks intended to violate its integrity. In our algorithm, a plain-image is substituted with a PRNG sequence (from the presented PRNG mechanism), while the proposed S-box mechanism is used to permutate each pixel of the substituted image, which combined coalesces as the encrypted image. Therefore, in addition to possessing key parameters for generating PRNG, the proposed algorithm is ingrained with key space needed for constructing the S-boxes (key parameters are used both for generating PRNG sequence and constructing S-box). Since both the PRNG sequence generation and S-box construction schemes are components of the proposed cascade quantum-inspired quantum walks on a circle and logistic-sine map technique, which both possess key parameters ($v,t,{\alpha }_{1},{\alpha }_{2},{\beta }_{1},{\beta }_{2},{x}_{0},\lambda$), then the key space for generating PRNG or constructing S-boxes is 10^128^ and, therefore, the key space allowance for the image encryption algorithm presented earlier is 10^256^, which is adequate for any encryption algorithm. Table 8 provides a comparison of key spaces for the proposed mechanism in comparison with similar approaches. Outcomes therefrom demonstrate our proposed mechanism has a superior key space allowance.

**Table 8:** Description of key space of our presented mechanism alongside those from similar methods.

Algorithm	Description	Key space
Proposed	Cascaded quantum walks as a quantum-inspired random generator and chaotic dynamics induction with its cryptographic applications	Key parameters (*v*, *t*, *α*_1_, *α*_2_, *β*_1_, *β*_2_, *x*_0_, *λ*) are utilised to run QWs and iterate logistic-sine map. The encryption algorithm is based on the presented PRNG mechanism and S-box mechanism. Therefore, the key space of whole system is 10^256^.
Yang *et al*.^45^	PRNG mechanism based on running 1-Dimensional 1-Particle quantum walks on a circle	Key parameters (*v*, *t*, *α*, *β*, *θ*) are utilized for running QWs. The key space for key parameters and initial states is 10^98^.
Yang *et al*.^25^	Image encryption algorithm based on running 1-Dimensional 2-Particle quantum walks on a circle	The key parameters (*v*, *t*, *α*_1_, *β*_1_, *α*_2_, *β*_1_, *θ*) are utilized for running QWs. The key space for key parameters is 10^98^.
Yang *et al*.^26^	Quantum hash function based on controlled 1-Dimensional 2-Particle quantum walks on a circle with its application to image encryption	Key parameters (*m*, *v*, *α*_1_, *β*_1_, *α*_2_, *β*_1_) are utilized for running QWs. The key space for key parameters and initial states is 10^98^.
Abd-El-Atty *et al*.^27^	Quantum greyscale image encryption algorithm based on controlled 1-Dimensional 1-Particle quantum walks on a circle	Key parameters (*m*, *v*, *t*, *α*, *β*, *θ*_1_, *θ*_2_, *θ*_3_) are used for running QWs. The key space of whole system is roundly 10^211^.

As suggested by the guideline in^52^ key space must be greater than ${2}^{100}\simeq {10}^{30}$ for it to exhibit sufficient security against brute-force attacks. In our case, the proposed approach has a key space of 10^256^ which consists of all possible keys. Consequently, to mitigate against the exhaustive search-attacks, a good cipher should have a key space size of $k > {10}^{98}$. This conforms with earlier guidelines in^25,26,45^. Based on the proposed approach, we can conclude that key size 10^256^ is adequate to forestall brute-force attacks in today’s and near future’s computers.

#### Key sensitivity analysis

**Figure 10:**
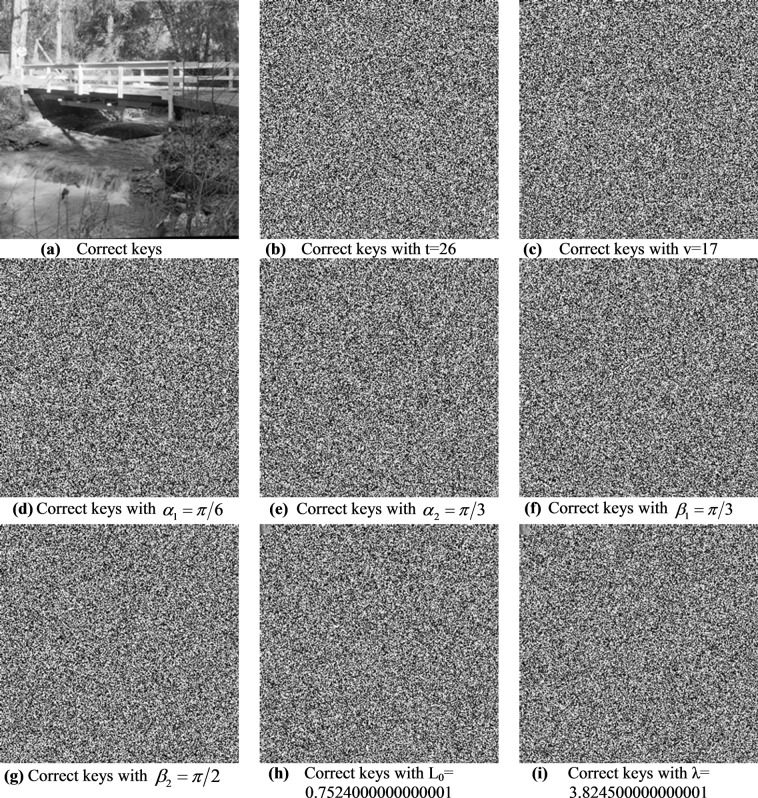
Decrypted Bridge image (in Fig. 3(g)) for several S-box keys.

**Figure 11:**
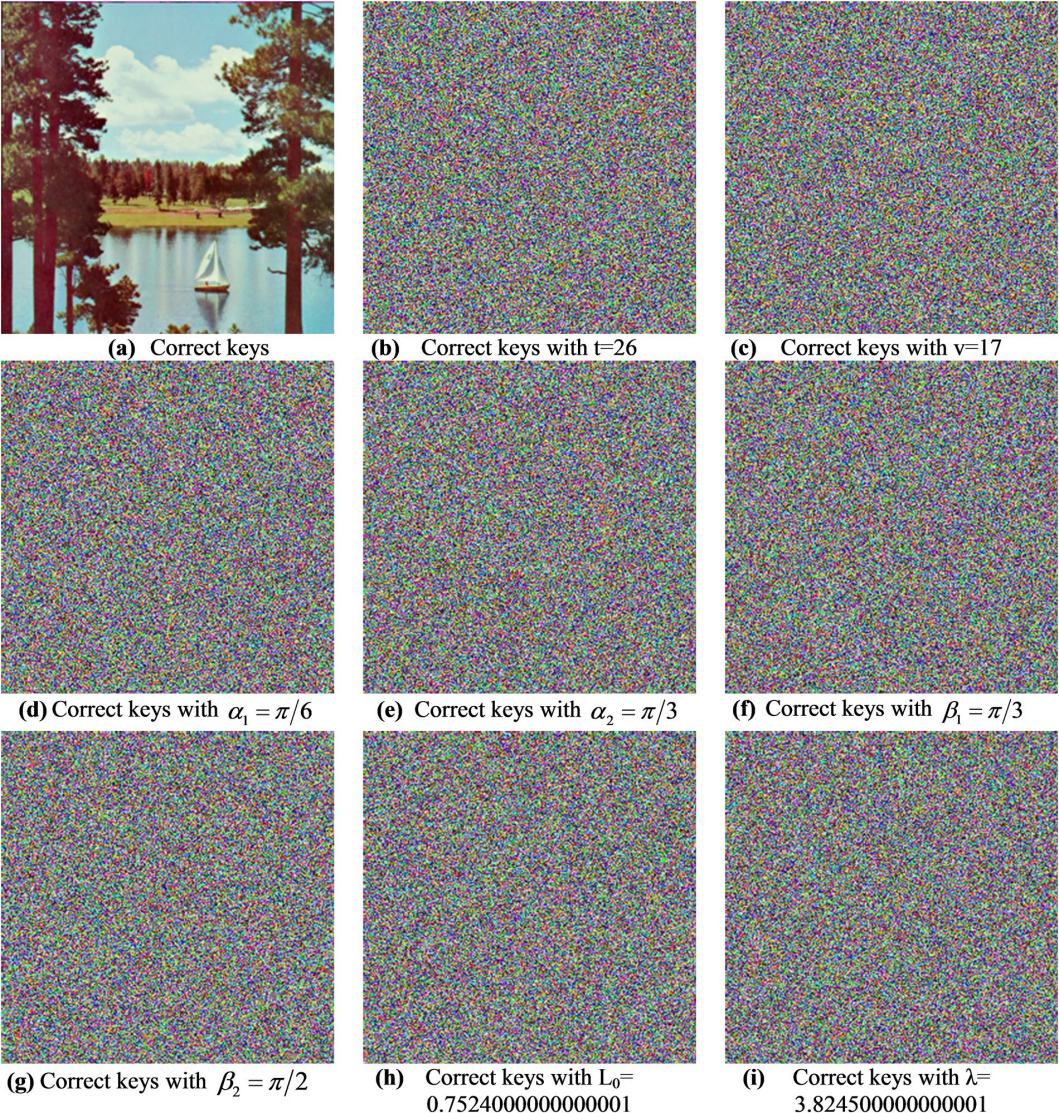
Decrypted Sailboat image for several PRNG keys.

To test the key sensitivity of the proposed image encryption algorithm, we demonstrate the decryption process for the encrypted Bridge and Sailboat images using several keys for constructing S-boxes and generating PRNG sequences. The results obtained therefrom are presented in Figs. 10 and 11, where Figs. 10(a) and 11(a) demonstrate near zero error during the scaling process for the probability matrix *P*.

## Discussion

Discrete-time quantum random walks are regarded as nonlinear mappings between quantum states and position probability distributions. They provide an imprint of chaotic behaviour, which are mathematical properties that can be exploited in constructing robust cryptographic applications. The study presented explores the potential for deploying quantum-inspired quantum random walks (QiQw) in the design of efficient cryptosystems. We have presented three quantum-inspired mechanisms that cascade quantum walks as a random number generators with logistic-sine map to ameliorate problems of periodicity in chaotic ranges, narrow key space and chaotic discontinuous ranges that are associated with traditional cryptosystems. First, we presented a mechanism for constructions of S-boxes with prospects for wide-ranging applications in security technologies. Second, we proposed a scheme to generate PRNG sequences that remedy the periodicity problem encountered in cryptographic applications. Third, we coalesced the two strategies into a cascaded quantum walks on a circle with logistic-sine map and implemented it as an image encryption algorithm. Based on simulations of our proposed schemes, we undertook extensive statistical analysis to validate the efficiency, reliability and utility of our proposed techniques alongside established methods employed in different cryptographic applications. With further improvements, the study presented provides useful insights to integrate state-of-the-art quantum-inspired quantum resources into building efficient, secure, and robust future cryptography technologies.

## Methods

Rudimentary background required for basic understanding of the proposed cascade quantum-inspired quantum walks and chaos system are highlighted in this section. Furthermore, a succinct overview on the execution of discrete-time quantum walks on a circle as well as the utility of logistic-sine map as a chaos system are expounded.

### Discrete-time quantum walks on a circle

Unlike in classical (i.e. digital or non-quantum) walks, the state of a quantum walk is a coherent superposition of several positions (quantum superposition of quantum walks)^53^, but much like their classical (i.e. digital) equivalents, there are two categories of quantum walks: discrete-time quantum walks and continuous-time quantum walks^20^. In this study, we focus on discrete-time quantum walks (or simply QWs), which have shown viability in wide-ranging cryptographic applications^18,19,25,26,28,30,45,54–58^. QWs have two basic parts: the walker space *H*_*p*_ and the coin particle ${H}_{c}=\,\cos \,\alpha |0\rangle +\,\sin \,\alpha |1\rangle$, which permeates a Hilbert space $|\psi {\rangle }_{0}={H}_{p}\otimes {H}_{c}$. The initial state of the system $|\psi {\rangle }_{0}$ can be transformed into another state via application of the evolution operator $\hat{U}$ for the whole quantum system

6 $$\hat{U}=\hat{S}(\hat{I}\otimes \hat{C})$$

where $\hat{S}$ refers to the shift operator that depends on the coin state of the particle, which can be defined on a circle with *v* vertices as presented in Eq. (7).

7 $$\hat{S}=\sum _{x}\,(|(x+1)\,{mod}\,v,0\rangle \langle x,0|+|(x-1)\,{mod}\,v,1\rangle \langle x,1|)$$

The operator $\hat{C}$ refers to a 2 × 2 coin operator, whose general case can be defined in (8).

8 $$\hat{C}=\left(\begin{array}{ll}\cos \,\beta  & \sin \,\beta \\ \sin \,\beta  & -\cos \,\beta \end{array}\right)$$

Hence, the final state $|\psi {\rangle }_{r}$ after *t* steps can be expressed as

9 $$|\psi {\rangle }_{t}={(\hat{U})}^{t}|\psi {\rangle }_{0}$$

The probability of finding the walker at position *x* after *t* steps can be stated as

10 $$P(x,t)=\sum _{c\in \{0,1\}}\,|{\langle x,c|{(\hat{U})}^{t}|\psi \rangle }_{0}{|}^{2},$$

where $|\psi {\rangle }_{0}$ is the initial state of the quantum system, $P(x,t)\in [0,1]$ and ${\sum }_{x=0}^{v}\,P(x,r)=1$.

Another attractive characteristic of multi-walker quantum random walks is that, in the case of interacting walkers, the dimension of the Hilbert space of an *n*-particle quantum walk (composed of distinguishable walkers) increases exponentially with the number of walkers, a property that supports increased entanglement. These properties are unattainable in classical random walks. Consequently, in our proposed model, the impetus for use of two instead of one quantum walker is its offer of increased keyspace allowance, which is crucial for designing efficient cryptosystems. Further details on interacting two quantum walks on a circle can be obtained from^19,59^.

In our proposed model of quantum walks, two coins $|coin{\rangle }_{1}$, $|coin{\rangle }_{2}$ and two walkers $|walker{\rangle }_{1}=\,\cos \,{\alpha }_{1}|0\rangle +\,\sin \,{\alpha }_{1}|1\rangle$, $|walker{\rangle }_{2}=\,\cos \,{\alpha }_{2}|0\rangle +\,\sin \,{\alpha }_{2}|1\rangle$ will be used. The combined shift operator for the system is $\hat{S}={\hat{S}}_{1}\otimes {\hat{S}}_{2}$^19,25,26^, where ${\hat{S}}_{1}$ and ${\hat{S}}_{2}$ are shift operators for $|walker{\rangle }_{1}$ and $|walker{\rangle }_{2}$, respectively. Following the same rationale, we shall use two coin operators, one for each coin $|coin{\rangle }_{1}$, $|coin{\rangle }_{2}$. Therefore, the combined coin operator is a Unitary operator that can be written as an order 4 matrix^19,25,26^. In this study, we have chosen the coin matrices presented in Eq. 11.

11 $$\hat{C}=\left[\left(\begin{array}{ll}\cos \,{\beta }_{1} & \sin \,{\beta }_{1}\\ \sin \,{\beta }_{1} & -\cos \,{\beta }_{1}\end{array}\right)\otimes \left(\begin{array}{ll}\cos \,{\beta }_{2} & \sin \,{\beta }_{2}\\ \sin \,{\beta }_{2} & -\cos \,{\beta }_{2}\end{array}\right)\right]$$

**Figure 12:**
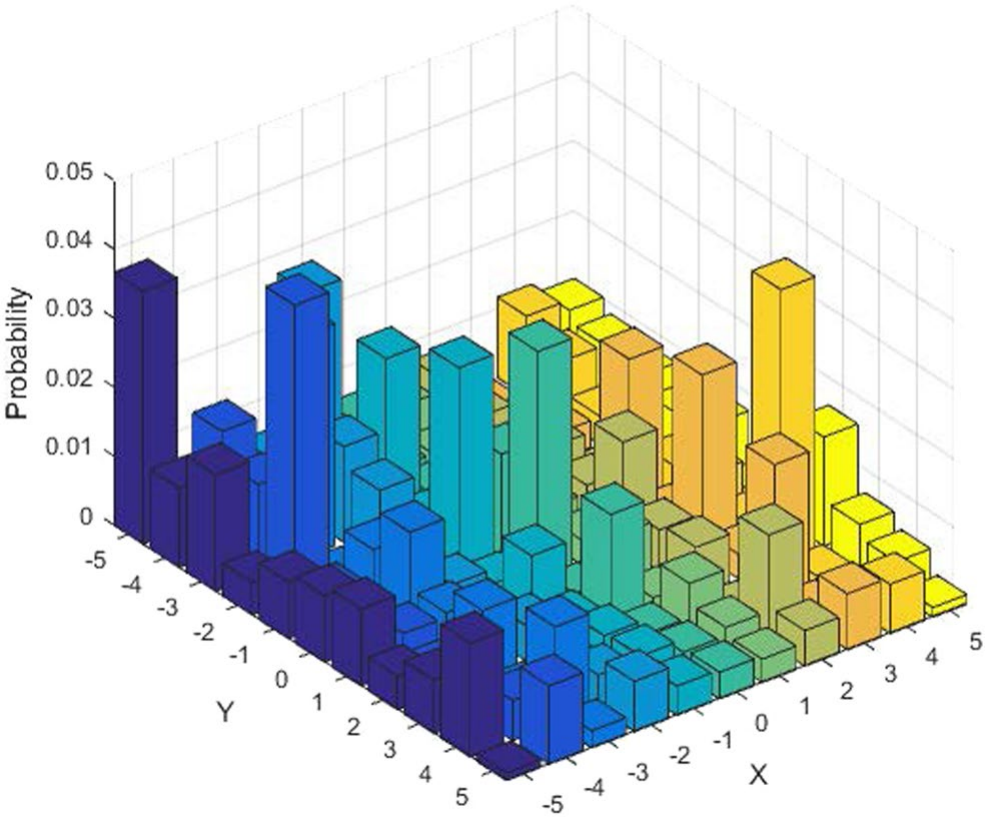
Probability distribution for running two-walker quantum walks on a circle with 11 vertices for 51 steps, where the initial coin particles are ${H}_{{c}_{1}}=|0\rangle$ and ${H}_{{c}_{2}}=|1\rangle$. Here, it is deducible that for a circle with only odd *v* nodes, the probability has nonzero in any position if the number of steps *t* is greater than the number of nodes *v*.

An example illustrating the probability distributions of running one-dimensional two-particle quantum walks on a circle with 11 vertices is presented in Fig. 12, where the initial position is $|0{\rangle }_{p}$ and the initial coin operator $\hat{C}$ constructed by ${\beta }_{1}=\pi /6$ and ${\beta }_{2}=\pi /3$ in formats stated in Eq. 11. It is obvious that, for a circle with only odd *v* nodes, the probability is nonzero in any position if the number of steps *t* is greater than or equal to the number of nodes *v*. In this study, we utilised the probability distribution generated from using quantum-inspired two-walker quantum walks in the cascading system whose construction is based on the coherent superposition of several positions of quantum walks rather than constructions from a mathematical model as obtains in chaotic maps. Like other quantum measurement operations, measurements to recover states of quantum walks, involve retrieval of probability distributions by repeating the measurement process many times, which is not completely accurate. Meanwhile, as clarified in our introductory commentary, our notion of quantum-inspired quantum walks entails the use of probability distributions that are obtained via numerical simulations using digital resources. Nevertheless, like any cryptographic mechanism, if the key parameters of the quantum-inspired quantum walk are disclosed, then anyone can access the probability distribution with appreciable precision. On the other hand, if the parameters are unknown, but a part of the probability distribution is disclosed, then it is very difficult to estimate the key parameters or the recover the probability distribution because our quantum-inspired quantum walk is a one-way mechanism^18,19,26^. Consequently, it is envisioned that the suggested cryptographic applications would offer additional layers of tamper-proof security within the precepts of quantum-inspired quantum walks.

### Discrete-time chaotic systems

As argued in earlier sections of this study, one-dimensional chaotic maps are considered in this study because they offer enhanced periodicity in chaotic ranges, narrow key space and chaotic discontinuous ranges when it is used in cryptographic systems^60^. However, the same one-dimensional chaotic maps exhibit powerful benefits in terms of high-speed processing, easy design and simple structure.

A widely used one-dimensional chaotic map is logistic-sine map^16^, which is expressed mathematically as

12 $${x}_{i+1}=(\lambda ({x}_{i}-{x}_{i}^{2})+(4-\lambda )\,\sin (\pi {x}_{i})/4)\,{mod}\,1$$

where $\lambda \in [0,4]$ is the control parameter, and *x*_0_ is the initial condition.

Depending on the set of times *T*, chaotic dynamical systems can be divided into two classes, i.e. either continuous-time dynamical system (i.e. when *T* = *R*) or discrete-time dynamical system (if T = Z). Our study focuses on applying chaotic dynamical systems defined in discrete time, since they possess low computational complexity and do not need synchronization as in continuous-time dynamical system^1–3^.
